# Monte Carlo simulation of spatial frequency domain imaging for breast tumors during compression

**DOI:** 10.1117/1.JBO.29.9.096001

**Published:** 2024-09-14

**Authors:** Constance M. Robbins, Kuanren Qian, Yongjie Jessica Zhang, Jana M. Kainerstorfer

**Affiliations:** a Carnegie Mellon University, Department of Biomedical Engineering, Pittsburgh, Pennsylvania, United States; b University of Pittsburgh, Department of Radiology, Pittsburgh, Pennsylvania, United States; c Carnegie Mellon University, Department of Mechanical Engineering, Pittsburgh, Pennsylvania, United States; d Carnegie Mellon University, Neuroscience Institute, Pittsburgh, Pennsylvania, United States

**Keywords:** spatial frequency domain imaging, Monte Carlo, finite element analysis, breast imaging, computational modeling

## Abstract

**Significance:**

Near-infrared optical imaging methods have shown promise for monitoring response to neoadjuvant chemotherapy (NAC) for breast cancer, with endogenous contrast coming from oxy- and deoxyhemoglobin. Spatial frequency domain imaging (SFDI) could be used to detect this contrast in a low-cost and portable format, but it has limited imaging depth. It is possible that local tissue compression could be used to reduce the effective tumor depth.

**Aim:**

To evaluate the potential of SFDI for therapy response prediction, we aim to predict how changes to tumor size, stiffness, and hemoglobin concentration would be reflected in contrast measured by SFDI under tissue compression.

**Approach:**

Finite element analysis of compression on an inclusion-containing soft material is combined with Monte Carlo simulation to predict the measured optical contrast.

**Results:**

When the effect of compression on blood volume is not considered, contrast gain from compression increases with the size and stiffness of the inclusion and decreases with the inclusion depth. With a model of reduction of blood volume from compression, compression reduces imaging contrast, an effect that is greater for larger inclusions and stiffer inclusions at shallower depths.

**Conclusions:**

This computational modeling study represents a first step toward tracking tumor changes induced by NAC using SFDI and local compression.

## 1 Introduction

Neoadjuvant chemotherapy (NAC) is commonly prescribed for locally advanced breast cancer, often shrinking the tumor prior to surgery and thereby permitting more breast-conserving surgeries or rendering operable some tumors initially too large to be operable. Pathologic complete response (assessed histologically after surgery) is strongly associated with prognosis, but methods to predict this response early in the course of therapy are lacking. Structural imaging modalities and clinical palpation can assess changes in tumor size but can fail to distinguish between fibrosis and residual disease.^1^^,^^2^ More success in outcome prediction has been demonstrated for dynamic contrast MRI,^3^^,^^4^ at the disadvantage of high cost (limiting frequent measurement) and the need for exogenous contrast agents.

Near-infrared (NIR) optical imaging has emerged as a promising method for NAC monitoring due to its relatively low cost, non-invasiveness, and sensitivity to endogenous contrast from hemoglobin concentrations.^4^^–^^16^ Increased total hemoglobin (tHb) relative to the background is thought to be related to angiogenesis within the tumor, and changes in vascularization in response to NAC are shown to occur earlier than structural changes,^4^^,^^5^ including the “oxy-hemoglobin flare” observed as early as 1 day after the start of NAC.^8^^,^^11^ Over the course of therapy, multiple groups have reported a decrease in tHb in responding tumors but not in non-responding tumors^10^^,^^17^^,^^18^ or a greater tHb decrease in responding tumors.^12^^,^^19^

Measuring the reaction of the breast to perturbations such as compression has also been used to provide additional contrast beyond that of baseline optical properties. A transient reduction in tHb of both tumor and healthy breast has been well documented in response to compression,^20^^–^^23^ with the change in tHb being related to pressure distribution within the breast.^24^ Carp et al.^23^ reported a gradual recovery of tHb in normal tissue while displacement is held constant (and force is reduced due to viscoelastic relaxation) while tHb of the tumor region remains persistently low. This hemodynamic response has also been demonstrated to normalize in patients who achieve response to NAC.^7^

To elicit this type of hemodynamic response as well as to longitudinally track tumor and background optical properties in a low-cost format, we are pursuing a handheld device for breast imaging with localized compression based on spatial frequency domain imaging (SFDI), a diffuse optical imaging method that uses structured illumination to quantify tissue absorption and reduced scattering coefficients.^25^^,^^26^ Because of its ability to quantify optical properties at a low cost and small form factor, the technique holds the potential for frequent monitoring during therapy, including in the patient’s home. As a reflectance-based 2D projection imaging method, SFDI suffers from partial volume effects, with superficial tissue contributing more strongly to the imaging result than deeper tissue.^27^ Previously, we showed in polydimethylsiloxane (PDMS) phantoms that compression can be used to reduce the depth of stiff highly absorbing inclusions and improve optical contrast measured with SFDI.^28^

We demonstrated that despite its high sensitivity to superficial tissue, SFDI can be used to detect tumor-mimicking inclusions at an initial depth comparable to palpable breast lesions and that localized compression can be used to increase the measured contrast. However, if longitudinal changes in contrast obtained with SFDI and local compression are to have utility as a biomarker for monitoring NAC, it is necessary to know what changes in imaging contrast would result from likely changes to tumor structure and function such as size,^29^^,^^30^ stiffness,^31^^,^^32^ and tHb concentration.^10^^,^^12^^,^^17^^–^^19^ We leverage a combination of mechanical and optical simulations to explore the relationship between these tumor parameters and optical contrast under compression.

One method for Monte Carlo (MC) simulation of SFDI involves the acquisition of a spatially resolved simulation of point source illumination (in the spatial domain) and the application of the discrete Hankel transform to convert it into the spatial frequency domain. By contrast, the Gardner method avoids the need for discrete transforms and performs the simulation natively in the spatial frequency domain by calculating a frequency-dependent photon weight.^33^ Both methods provide diffuse reflectance over a range of spatial frequencies and are suitable for homogenous or layered input media. However, neither can generate spatially resolved optical property maps of a complex 3D geometry.

To simulate the result of SFDI on an embedded highly absorbing inclusion (mimicking breast tumor), we employed MC to directly simulate the projection of a sinusoidal modulated light source at three phase shifts. The results are demodulated to obtain 2D diffuse reflectance maps, which are converted to optical property maps using a two-frequency lookup table (LUT) in the conventional manner for SFDI. In this method, diffuse reflectance information is limited only to those spatial frequencies that are simulated individually at three phases each, representing a considerable simulation run time. In addition, many photons must be launched for each MC simulation to limit noise in the detected reflectance with too few resulting in error in demodulation of the sinusoidal patterns. For this reason, only a single spatial frequency, $0.1\;{\mathrm{mm}}^{-1}$, is simulated at three phases, from which DC reflectance of $0\;{\mathrm{mm}}^{-1}$ is also extracted according to the demodulation equations given in Gioux et al.^26^ This spatial frequency pair was chosen to be consistent with that used in our group’s handheld SFD breast imager^34^ and has been established to be sufficient for decoupling absorption ($\mu_{a}$) and reduced scattering coefficients ($\mu_{s}^{\prime }$).^35^

To predict the final imaging result, we must model not only the 2D SFDI reflectance image that would arise from each input but also the change in tumor shape and depth from compression and the effect of compression on final optical properties in the tissue input. Finite element analysis (FEA) has been adopted together with image processing techniques to study tumor growth,^36^ tracking,^37^ and treatment.^38^^,^^39^ In this study, FEA via ANSYS mechanical is used to predict the change in tissue geometry resulting from compression. Subsequent spatially resolved MC simulations are performed, both with and without modeling tissue blanching due to compression. Tissue blanching is modeled as occurring in all tissue, termed the “full blanching” condition, or as occurring in the background but not inclusion, the “background blanching” condition, as the internal stresses within the enclosed environment of tumors may prevent blood volume reduction from occurring in the same manner as healthy tissue.

## 2 Methods

**Fig. 1:**
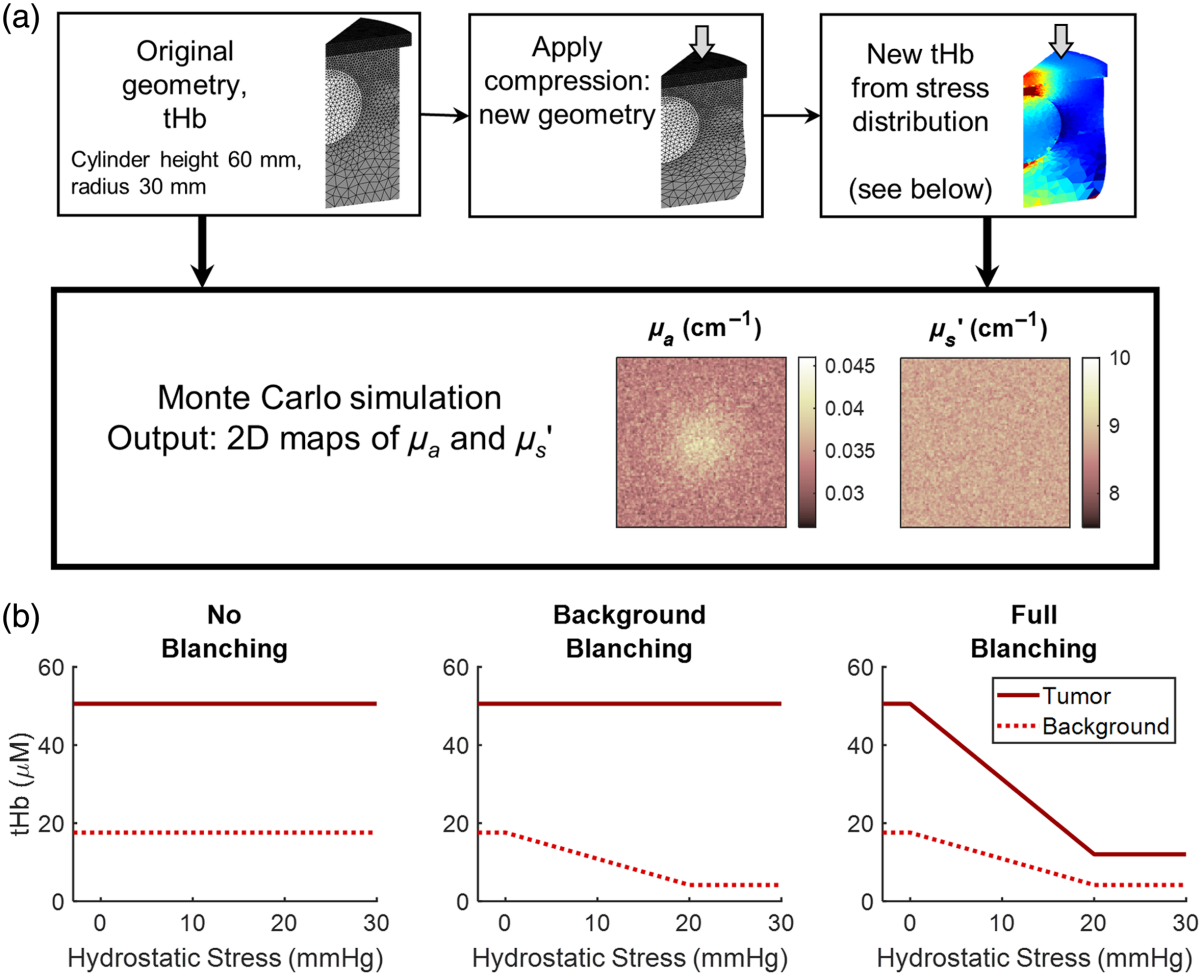
(a) Summary of simulations performed. (b) Following mechanical simulations, tHb concentrations are adjusted on a voxel-by-voxel basis based on hydrostatic stress experienced by the model. The three models for the dependence of hemoglobin concentration on stress are illustrated here. In the “no blanching” model, tHb is held constant in both the inclusion and background regions. In the “background blanching” model, tHb of inclusion remains constant while tHb of the background region decreases linearly before plateauing at 20 mmHg. In the “full blanching” model, both inclusion and background regions experience a decrease in tHb with experienced pressure.

The effect of tissue compression on the output of SFDI was examined across various tumor sizes, depths, and stiffness ratios (SRs). A breast tumor and surrounding healthy tissue were modeled as a stiff, spherical inclusion embedded in a softer cylindrical matrix, with optical properties based on typical tumor and healthy breast values. The deformation of this model under compression was simulated using ANSYS Mechanical, with new optical properties calculated based on the stress output of the mechanical simulation. MC SFDI simulations were then performed using Monte Carlo eXtreme (MCX).^40^ This workflow of mechanical and optical simulations is illustrated in Fig. 1(a).

MC simulation is performed four times for each tissue input: one on the original geometry before compression and three on the compressed geometry corresponding to three models of the change in tHb concentration with pressure. In the “no blanching” condition, optical properties for inclusion and background remain equal to pre-compression values. In the “background blanching” and “full blanching” conditions, new optical properties are calculated on a voxel-by-voxel basis based on the stress distribution in the model for the background only and for both regions, respectively. The dependence of tHb on stress experienced is shown in Fig. 1(b). Results of the MC simulations were compared to the initial inclusion radius, $r_{0}$, and depth, $d_{0}$, and to the mid-compression surface radius of curvature, r, and mid-compression depth, d.

### 2.1 Mechanical Simulation

For all mechanical simulations, a Young’s modulus of 4 kPa was used for the background material, based on results reported by Samani et al.^41^ for healthy breast. In that study, a Poisson’s ratio value of 0.495 was used in their FEA simulations, and the same value was used here. To mimic the stiffness contrast of breast tumors to healthy breast,^41^ the inclusions were given elastic moduli of 4, 16, 28, and 40 kPa, resulting in SRs of 1, 4, 7, and 10. The mechanical model consisted of a cylinder of background material of height 60 mm and radius 30 mm with the spherical inclusion located along the center axis of the cylinder, a variable distance from the top surface of the cylinder. Inclusion depths simulated were 3, 5, 7, 10, and 15 mm (measured from cylinder surface to inclusion surface) and inclusion radii simulated were 7.5, 12.5, 15, and 17.5 mm. A fixed boundary condition was applied to the cylinder bottom (opposite of the face to be imaged). A rigid plate (modeled as structural steel: Young’s modulus of $2\times 10^{8}\;\mathrm{kPa}$, Poisson’s ratio of 0.3, and density of $7850\;\mathrm{kg}\cdot m^{-3}$) was placed on the top surface of the cylinder (face to be imaged) with a frictional boundary condition with the cylinder and a friction coefficient of 0.1. This friction coefficient was intended to approximate the friction between the glass imaging head of the handheld SFDI device and the patient’s skin and was assumed not to differ greatly from the friction of lubricated glass on glass.^42^ To reduce the computational load, symmetry was used to reduce the size of the model, as is common in the field.^43^ Only a 1/8 slice was simulated instead of the entire cylinder, with a frictionless support boundary condition applied to the faces cut through the cylinder. An upward displacement of 12.5 mm was applied to the bottom surface of the cylinder. These simulations took between 10 and 35 min each, for a total computation time of ∼24  h.

### 2.2 Processing of FEA Output

For all FEA outputs, as well as the undeformed input meshes, the 1/8 slice was mirrored to form a full cylinder. The boundary surface between the inclusion and background nodes was extracted, as was the top surface of the background cylinder. The “iso2mesh” MATLAB toolbox was used to create a labeled volumetric image from these boundaries. The volumetric image had dimensions of 80×80  mm for the top face and a depth of 62.5 mm (192×192×150  voxels). Background-labeled voxels were extended out to the edges of the rectangular slab regardless of the outer surface of the FEA cylinder. The inclusion depth was extracted from the volumetric images for both compressed and uncompressed (UC) tissue inputs as the voxelization of the image caused depth to vary slightly from the inclusion depth of the FEA mesh. Similarly, the radius of curvature of the top surface of the inclusion was extracted from the volumetric images. A cross-section of the image through the center of the inclusion (plane orthogonal to the imaging surface) was taken, and the boundary pixels were identified with the MATLAB’s “bwboundaries” function. A circle was fit limited to those points located on the upper one-third of the inclusion.^44^

Initial (pre-compression) tHb concentrations, tHb, for the main simulations were defined to be 50.6  μM for tumor voxels and 17.6  μM for background voxels, within the ranges reported by Grosenick et al.^5^ Simulations were also performed for tumor tHb of 63.3  μM (a 25% increase) and background voxels remaining at 17.6  μM, to verify the effect of increasing intrinsic tumor absorption contrast. The absorption coefficient at 805 nm (close to the isosbestic point) was calculated for each based on the extinction spectrum of hemoglobin^45^ and assuming 75% tissue oxygen saturation. Though other chromophores are present in the tissue, including water, collagen, and lipid, their contribution in this region of NIR is small,^46^^,^^47^ and only light absorption for tHb was considered. The resulting $\mu_{a}$ was $0.0329\;{\mathrm{cm}}^{-1}$ for background, $0.0948\;{\mathrm{cm}}^{-1}$ for tumor, and $0.1185\;{\mathrm{cm}}^{-1}$ for tumors with increased tHb. For all simulations, $\mu_{s}^{\prime }$ was defined to be $10.37\;{\mathrm{cm}}^{-1}$ for tumor voxels and $8.72\;{\mathrm{cm}}^{-1}$ for background voxels.^5^ MC simulations of UC models were run using the specified baseline values for inclusion and background voxels. MC simulations were also run on all compressed geometries maintaining these initial optical properties for tumor and background voxels, with these results deemed the “no blanching” condition. The purpose of this condition was to model the effect of compression on change in optical contrast that arises from the change of shape and depth of a highly absorbing inclusion.

MC simulations were then run for the two tissue blanching conditions, in which the change in tHb concentration of the tissue is modeled based on the spatially varying stress experienced by the tissue input. FEA output stress was also converted to a volumetric image of the same dimensions as the tissue input. Hydrostatic stress was obtained by averaging the three normal stresses at each node. Hydrostatic stress at the center of each voxel was interpolated via MATLAB’s “scatteredinterpolant” function. Because the stress was undefined outside the cylinder boundary, stress located ∼1.5  mm inside the cylinder boundary was extrapolated outward to the edge of the volumetric image. Stress in the bottom 15 mm of the image was defined to be zero. As these voxels are far from the surface to be imaged, they contribute minimally to the diffuse reflectance. Hydrostatic stress values were converted to mmHg for ease of comparison with typical pressure in blood vessels.

A piecewise function was defined to model tissue blanching in response to pressure. Blood volume in the venous compartment (including veins and venules) was assumed to comprise 76.2% of systemic blood volume and to contain a maximum blood pressure of 20 mmHg.^48^ For pressures between 0 and 20 mmHg, blood volume in the venous compartment was modeled to decrease linearly. Above 20 mmHg, the venous compartment was considered fully collapsed due to the lower pressure within veins,^48^ and thus, no further decrease in blood volume occurred after 20 mmHg. For pressures below zero, the venous compartment maintains its original volume. As the compression pressures studied were below arterial pressure, no effect on the arteries was modeled.

Mid-compression tHb was calculated on a voxel-by-voxel basis using the function defined above and the corresponding hydrostatic stress matrix, and $\mu_{a}$ at 805 nm (close to the isosbestic point) was calculated based on the extinction spectrum of hemoglobin.^45^ For the “background blanching” condition, new $\mu_{a}$ was calculated only for background voxels with the inclusion voxel optical properties remaining at pre-compression values. In the “full blanching condition, new $\mu_{a}$ was calculated for all voxels (both inclusion and background). The effect of compression on $\mu_{s}^{\prime }$ was not modeled.

### 2.3 Implementation of Monte Carlo Simulations

For each input, three MC simulations were performed corresponding to three evenly spaced phase shifts of the spatially modulated light pattern, with all other simulation parameters held constant. Outputs from the three simulations were demodulated on a pixel-by-pixel basis in a fashion similar to conventional SFDI and consistent with our group’s handheld SFD breast imager. In our in vivo imaging of breast hemodynamics, projection of only one spatial frequency, from which reflectance at $0\;{\mathrm{mm}}^{-1}$ is also extracted, allows fast image acquisition and fast processing of results. Likewise, simulation of one non-zero spatial frequency provides the same benefit here, while allowing the most consistency of methods with our *in vivo* SFDI results. The pair of 0 and $0.1\;{\mathrm{mm}}^{-1}$ was chosen for both as it has been established to be sufficient for decoupling $\mu_{a}$ and $\mu_{s}^{\prime }$.^35^ Although lower spatial frequencies have been shown to provide higher sensitivity to deeper tissue,^27^ lower frequencies do not allow for as efficient separation of $\mu_{a}$ and $\mu_{s}^{\prime }$ when paired with $0\;{\mathrm{mm}}^{-1}$.^35^

The sinusoidal spatially modulated source was defined using the MCX source type “fourier” with spatial frequency $f_{x}=0.1\;{\mathrm{mm}}^{-1}$ and phase shifts of 0, 2π/3, and 4π/3. Cyclic boundary conditions (photons exiting from one face renter from the opposite face) were used for the side faces to mimic an infinitely tiled medium. Default boundary conditions were used for the remaining two, with photons counted as diffuse reflectance when escaping through the source-incident face and lost when exiting through the opposite face. With a total depth of 60 mm, a negligible amount of photons are lost through the bottom surface. A refractive index, n, of 1.4 was used for background and inclusion material as this is a commonly used estimate for biological tissue.^49^^,^^50^ A refractive index value of 1 was used for the region outside the sample, representing air. For the anisotropy factor, g, a value of 0.8 was used, within the range of values reported in the literature.^27^^,^^51^^,^^52^

The raw output flux at each pixel of the tissue/air interface (x,y) was demodulated to yield 2D maps of AC and DC flux. Equations of demodulation are given as:

$$\varphi_{\mathrm{DC}}=(\varphi_{1}+\varphi_{2}+\varphi_{3})/3,$$ (1)

$$\varphi_{\mathrm{AC}}=\frac{\sqrt{2}}{3}\cdot \sqrt{(\varphi_{1}-\varphi_{2}{)}^{2}+(\varphi_{2}-\varphi_{3}{)}^{2}+(\varphi_{3}-\varphi_{2}{)}^{2}},$$ (2)

where $\varphi_{1}$, $\varphi_{2}$, and $\varphi_{3}$ are the raw output flux of the three source phase shifts. This demodulation is performed in the same manner as the demodulation of intensities $I_{1}$, $I_{2}$, and $I_{3}$ in conventional SFDI processing.^53^ The distinction is that those intensities are of arbitrary units and must be compared with intensities obtained from a calibration phantom to obtain $R_{d}$, whereas these simulation outputs represented photon flux with units of $\frac{1}{{\mathrm{mm}}^{2}\cdot s}$, and for that reason, they are denoted φ rather than I. $\varphi_{\mathrm{DC}}$ and $\varphi_{\mathrm{AC}}$ were then multiplied by timestep and area of the source-incident surface to produce unitless diffuse reflectances, $R_{d,\mathrm{DC}}(x,y)$ and $R_{d,\mathrm{AC}}(x,y)$. Each pixel value of the diffuse reflectance maps is within zero and one and represents the fraction of light that is diffusely reflected at that location.

An MC LUT was generated by running simulations at three phases of the sinusoidal light input with $f_{x}=0.1\;{\mathrm{mm}}^{-1}$ for every combination of $\mu_{a}$ from 0.01 to $0.28\;{\mathrm{cm}}^{-1}$ (increments of $0.01\;{\mathrm{cm}}^{-1}$) and $\mu_{s}^{\prime }$ from 4.0 to $20\;{\mathrm{cm}}^{-1}$ (increments of $1.0\;{\mathrm{cm}}^{-1}$). For the LUT simulations, a homogenous slab of 24×24×150  voxels (10×10×62.5  mm) was used. A “cyclic” boundary condition was defined for the four edge faces such that when a photon escaped from one side, it would re-enter the slab from the opposite face. This boundary condition allowed the simulation to mimic an infinitely wide slab with a relatively short computational time. A “total absorption” boundary condition was used for the bottom face as it was determined that a negligible number of photons reached the bottom surface of the slab. For each pair of optical properties, $1\times 10^{7}\text{ photons}$ were launched for each of the three source phases. $R_{d,\mathrm{DC}}$ and $R_{d,\mathrm{AC}}$ were averaged across the entire image and stored for each optical property combination. This quantity of photons launche was determined to be sufficient from visual inspection of the isolines of the resulting LUT, which were observed to be smooth.

MC simulations of the compressed and UC tissue geometries differed from the LUT simulations only in the tissue input and the number of photons launched. $1.4\times 10^{8}\text{ photons}$ were simulated per phase. More than an order of magnitude greater number of photons was used due to the larger tissue input and because spatial maps of optical properties were to be calculated, necessitating a lower signal-to-noise ratio than the LUT simulations in which $R_{d,\mathrm{DC}}$ and $R_{d,\mathrm{AC}}$ were averaged over the image. The tissue input definition was 192×192×150  voxels (80×80×62.5  mm) and had continuously varying optical properties as determined above. All MC simulations of the UC tissue input as well as the “no blanching” and “full blanching” conditions took ∼33  h to complete. Simulations for the “background blanching” condition were performed later with higher computational power and required ∼2  h, collectively. Spatially varying optical properties were calculated from diffuse reflectance pixel-by-pixel using the LUT created above and MATLAB’s “scatteredinterpolant” function, yielding $\mu_{a}(x,y)$ and $\mu_{s}^{\prime }(x,y)$.

### 2.4 Processing of MCX Output

Optical contrast was quantified by subtracting the baseline value from the $\mu_{a}$ map and summing the resulting pixel values over the area formed by projecting the inclusion boundary onto the surface. Though this sum would result in units of ${\mathrm{cm}}^{-1}$, optical contrast was instead treated as having arbitrary units. For all conditions, the baseline $\mu_{a}$ value was extracted from the final $\mu_{a}$ map by averaging all pixels located at the image margins, at least 45 mm from the inclusion epicenter, at which location the inclusion contributes minimally to the result. This region of interest (ROI) was determined empirically by comparing the image margins of the UC simulation with the highest contrast and a case with no detectable contrast (the smallest inclusion at the deepest depth), and the image margins were found not to differ.

Though contrast calculated with the above method was used for most analyses, the tumor/normal (T/N) ratio was also calculated as this is a common measure in breast cancer optical imaging. In addition, a threshold of 5% difference from baseline (T/N ratio below 0.95 or above 1.05) was used to determine which results were considered detectable, as this level agreed with a visual inspection of the $\mu_{a}$ maps.

## 3 Results

### 3.1 Results without Change in tHb (“No Blanching” Condition)

**Fig. 2:**
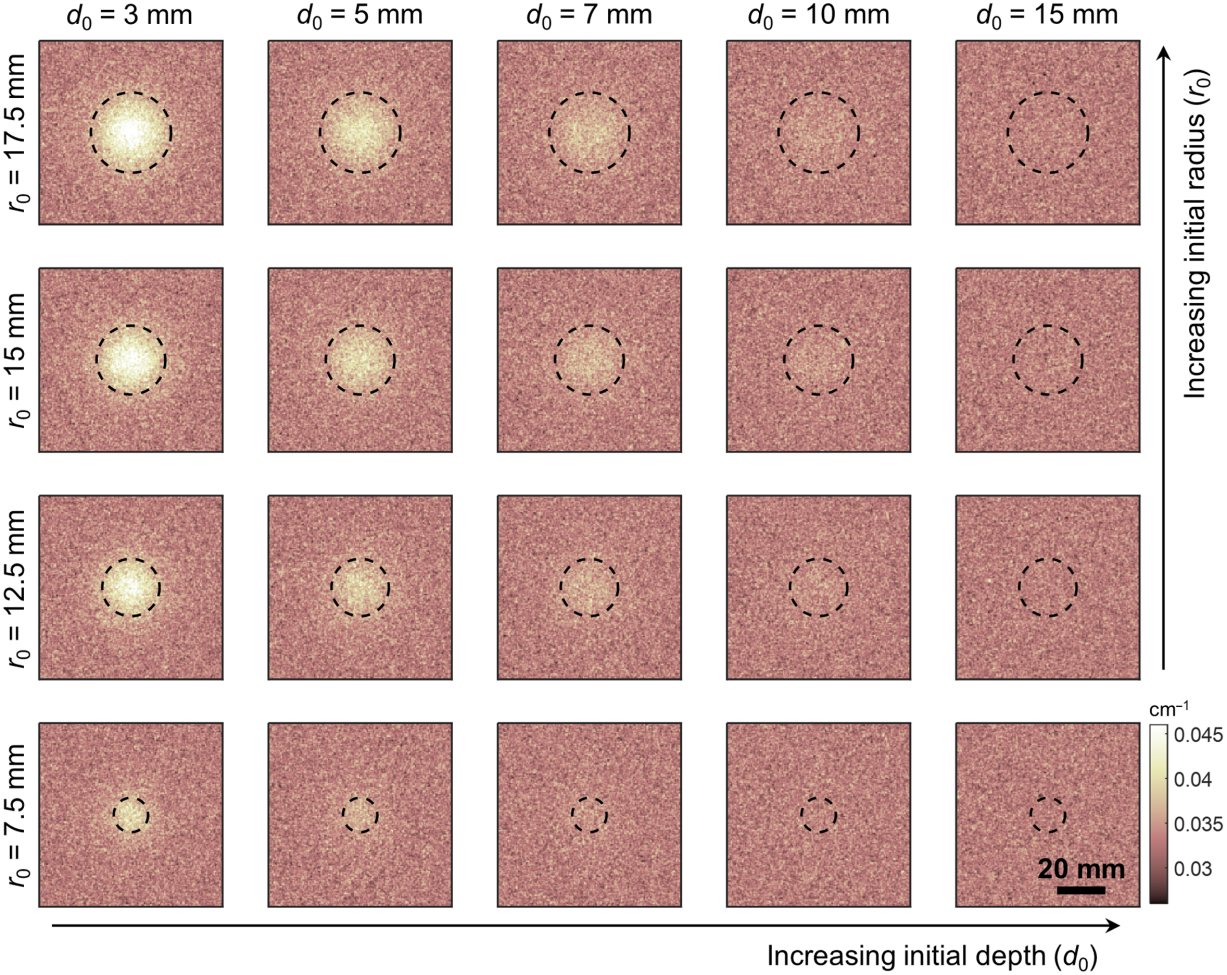
Summary of MC simulation results for UC tissue input. Each square corresponds to the $\mu_{a}$ output for the inclusion radius, $r_{0}$, and depth, $d_{0}$, indicated. Dotted circles represent the projection of the inclusion outline onto the imaging surface.

MC-derived $\mu_{a}$ maps of UC geometry show the expected trends with depth and radius: larger and shallower inclusions having the highest contrast over the background. These maps are shown in Fig. 2, with the highest contrast in $\mu_{a}$ evident in the upper left corner ($r_{0}=17.5\;\mathrm{mm}$, $d_{0}=3\;\mathrm{mm}$). Moving to the right (corresponding to greater inclusion depth) or down (corresponding to smaller inclusion radius) in Fig. 2 both yield lower levels of contrast in $\mu_{a}$. All figures refer to the results using 50.6  μM tHb for tumor and 17.6  μM tHb for healthy breast.

**Fig. 3:**
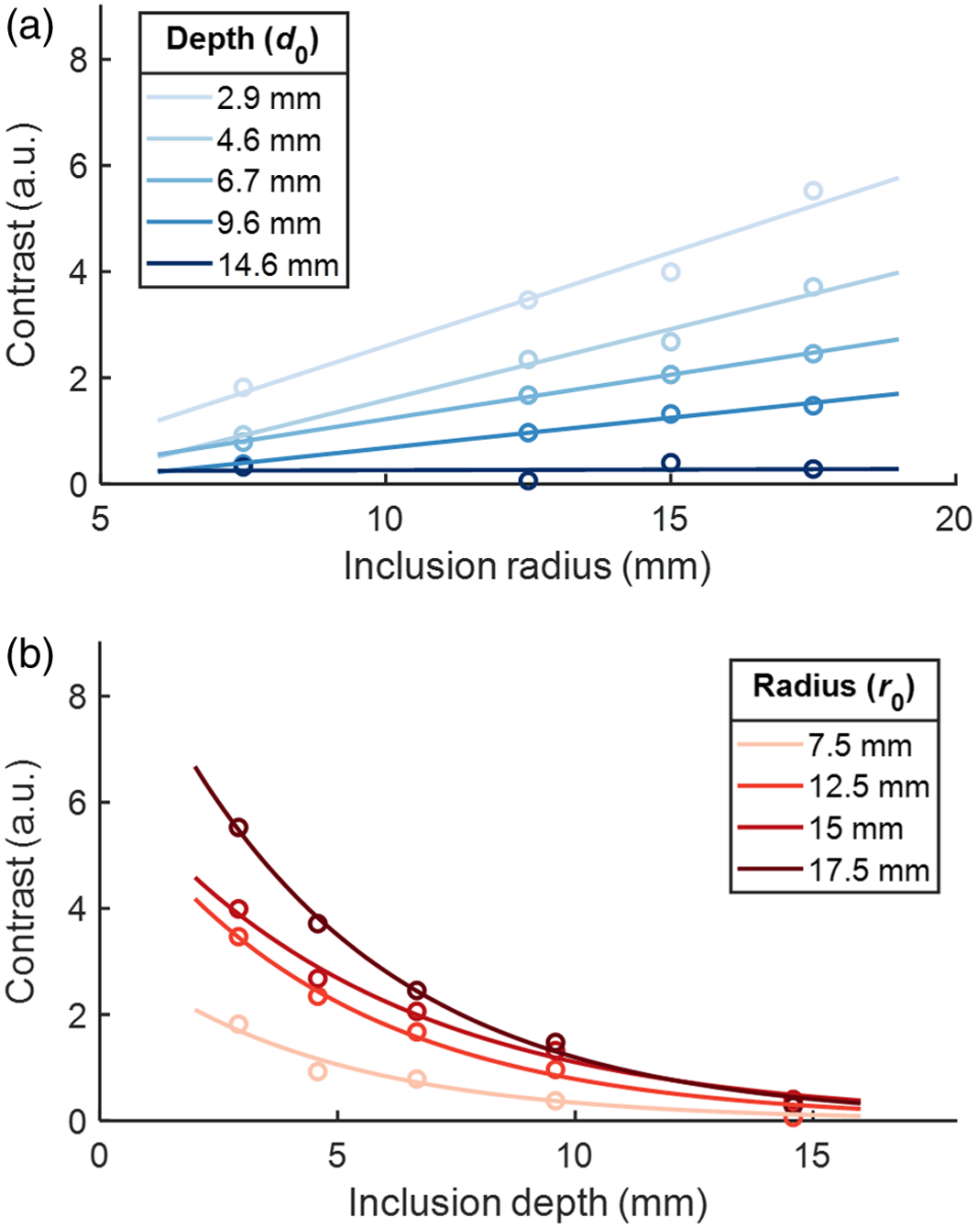
Relationship of simulation output contrast to initial inclusion radius, $r_{0}$, and depth, $d_{0}$. (a) When depth is held constant, contrast is found to increase linearly with increasing radius. (b) When the inclusion radius is held constant, contrast is found to decrease exponentially with increasing depth. Plot colors adapted from Ref. 54.

When optical contrast is computed by baseline subtraction and summation of $\mu_{a}$ over the area, these relationships can be quantified. When depth is held constant, contrast is found to have a linear relationship with inclusion radius, shown in Fig. 3(a). Equations of best fit of the form $C=a\cdot r_{0}+b$ are obtained for these linear relationships, where C represents contrast and $r_{0}$ represents the initial radius. Coefficients are given in Table 1.

**Table 1:** Summary of the relationship between contrast and initial radius when depth is held constant.

Initial depth ($d_{0}$) (mm)	a	b
2.9	0.407	−0.598
4.6	0.267	−0.110
6.7	0.178	0.183
9.6	0.099	0.433
14.6	0.037	0.581

Equations of best fit are of the form $C=a\cdot r_{0}+b$.

When the radius is held constant, contrast is found to have an exponential relationship with inclusion depth, shown in Fig. 3(b). Equations of best fit of the form $C=a\cdot e^{b\cdot d0}$ are obtained for these exponential relationships, where C represents contrast and $d_{0}$ represents initial depth. Coefficients are given in Table 2.

**Table 2:** Summary of the relationship between contrast and initial depth when the radius is held constant.

Initial radius ($r_{0}$) (mm)	a	b
7.5	3.11	−0.099
12.5	6.53	−0.142
15	8.50	−0.158
17.5	10.1	−0.162

Equations of best fit are of the form $C=\;a\;\cdot \;e^{b\cdot d_{0}}$.

This exponential decrease with depth is consistent with previous findings for PDMS phantoms with stiff, highly absorbing inclusions.^28^

**Fig. 4:**
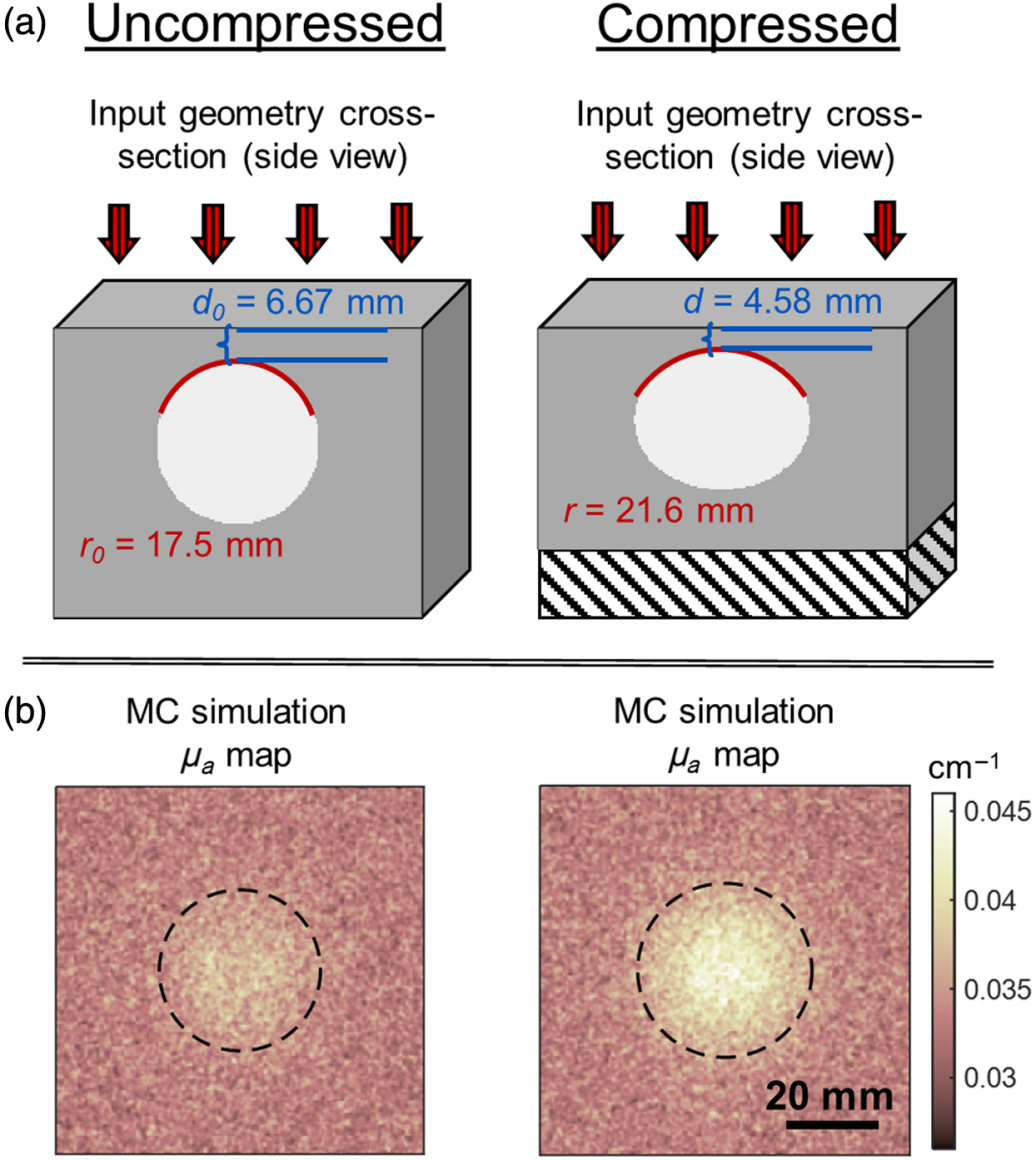
Compression increases the inclusion radius of curvature and decreases depth, resulting in increased contrast. (a) Illustration of the effect of compression on input geometry. The black and white striped area indicates the phantom height reduction from compression. (b) Results of MC simulations on UC and compressed conditions. Dotted lines mark the outline of the inclusion projected onto the phantom surface.

The effect of compression in all cases is to decrease the final depth compared to the initial depth ($d<d_{0}$) and to increase the top surface radius of curvature compared to the initial radius ($r>r_{0}$). Thus, when the optical properties of the inclusion and background are held constant with compression, optical contrast is gained from compression in all but two cases. The two cases that did not experience an increase in contrast consisted of the smallest and deepest inclusions with negligible contrast (T/N ratio between 1.0 and 1.05) both before and after compression. A representative example of the effect of compression on the initial geometry and the resulting change in MC-derived $\mu_{a}$ is shown in Fig. 4. The effect of compression on the inclusion depth and radius of curvature is shown in the cross-sections of Fig. 4(a), and the resulting simulated $\mu_{a}$ maps are shown in Fig. 4(b), with increased contrast evident for the compressed simulation. In this example, the initial radius was 17.5 mm, and the initial depth was 6.67 mm, with SR=2. After compression, the radius of curvature increased to 21.6 mm, and depth decreased to 4.58 mm. Background voxels were $\mu_{a}=0.0329\;{\mathrm{cm}}^{-1}$, and inclusion voxels were $\mu_{a}=0.0948\;{\mathrm{cm}}^{-1}$

**Fig. 5:**
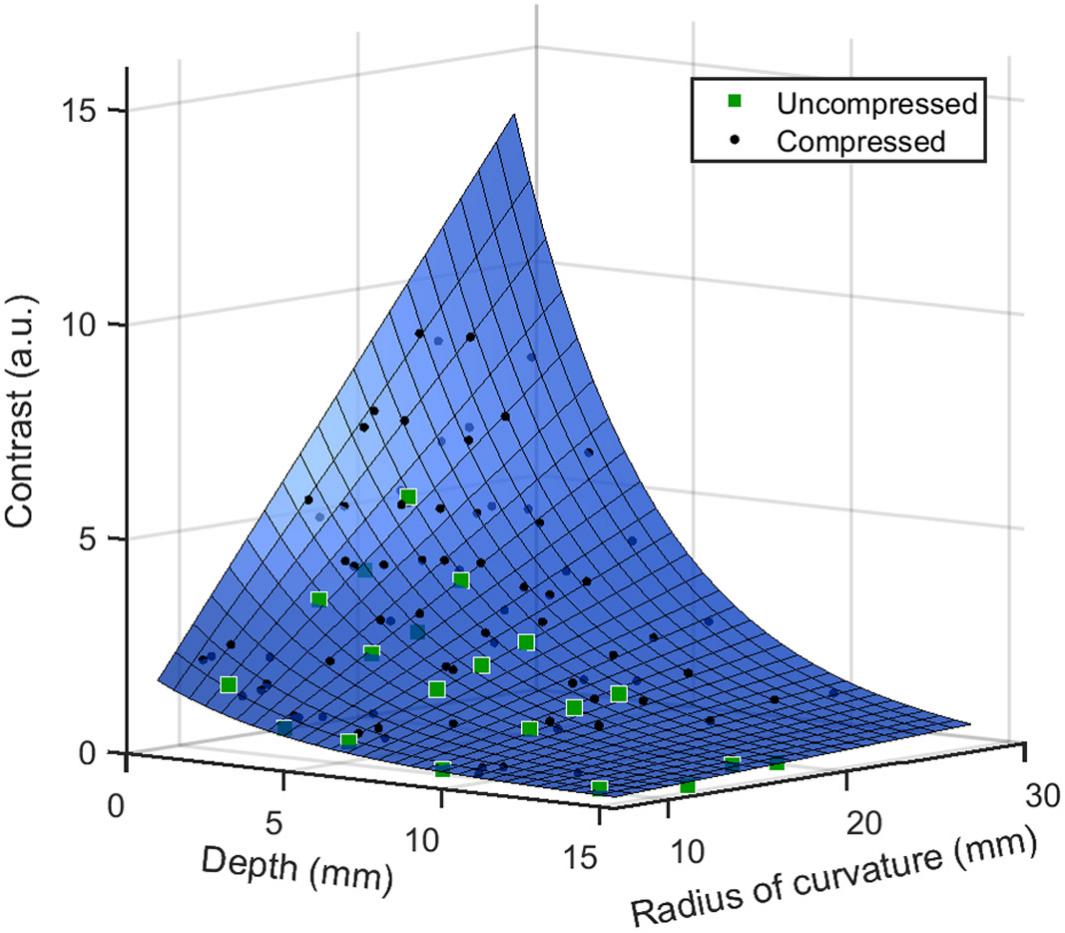
Relationship of the final radius of curvature, r, final depth, d, and contrast for the solid phantom condition. Green squares indicate simulations of the 20 UC geometries ($r=r_{0}$ and $d=d_{0}$), and black circles represent the 80 compressed simulations (20 inputs at 4 SR values).

Just as contrast before compression bore a strong relationship to $r_{0}$ and $d_{0}$, contrast during compression is strongly related to final geometric parameters, r and d. Though the value of these properties during compression is not expected to be known in a potential clinical application of this work, it is of interest to quantify how the values of r and d affect optical contrast in this study to characterize the effect of compression-induced changes in shape and depth on optical contrast. Because r and d do not occur at discrete intervals as $r_{0}$ and $d_{0}$ do, all points were plotted on a set of 3D axes (with contrast on the z-axis) instead of examining the relationship when each parameter is held constant. When contrast is plotted as a function of r and d, all points are found to lie within the same surface in 3D space, as shown in Fig. 5.

The equation

$$z=(c_{1}+c_{2}y)\cdot e^{c_{3}x}+c_{4},$$ (3)

describing this surface, was obtained via least squares fitting (MATLAB’s “fit” function), where y is the radius, x is the depth, z is the contrast, and $c_{1}$, $c_{2}$, $c_{3}$, and $c_{4}$ are constants obtained by the fit. This form was chosen to account for the previously observed linear relationship with radius when depth is held constant and the exponential relationship with depth when radius is held constant. The equation of the best-fit surface was $z=(0.802+3.694r)\cdot e^{-0.2259d}0.8871$. This surface describes a high degree of variance in contrast, with $R^{2}=0.992$. This goodness of fit is also apparent from the visual inspection of Fig. 5. Thus, the SR is not necessary for the prediction of final contrast if the final depth and radius of curvature are known. This result also demonstrates that the top surface radius of curvature is a sufficient description of the shape and size of a deformed spherical inclusion. For this type of imaging, a flattened ellipsoidal inclusion mimics a larger spherical inclusion as only the top surface contributes significantly to optical contrast.

We have demonstrated that final contrast is determined only from final geometry parameters r and d. However, in a potential clinical application of this work, mid-compression r and d are not expected to be known, though it is of interest to predict contrast change (difference between final and initial contrast) as a function of the input parameters $r_{0}$, $d_{0}$, and SR. All simulation cases in the “no blanching” condition exhibited an increase in contrast induced by compression, with the exception of two cases of small and deep inclusions, which exhibited negligibly low contrast values both before and during compression. These results are visualized below in Sec. 3.3 along with the two tissue blanching conditions.

When simulations are repeated with tHb concentration in the tumor voxels increased from 50.6 to 63.3  μM tHb, the same trends as above are found. The initial radius still exerts the most influence on contrast change, followed by initial depth and a weak influence from the SR. When the 63.3  μM simulations are compared directly with the 50.6  μM simulations, those with greater hemoglobin concentration in the tumor voxels experience greater contrast gain from compression, other variables being equal (not shown). This finding is consistent with the intuition that, as tumor voxels are brought close to the surface via compression, more highly absorbing tumor voxels will cause a greater gain in imaging contrast.

### 3.2 Results with Change in tHb (“Background Blanching” and “Full Blanching” Conditions)

**Fig. 6:**
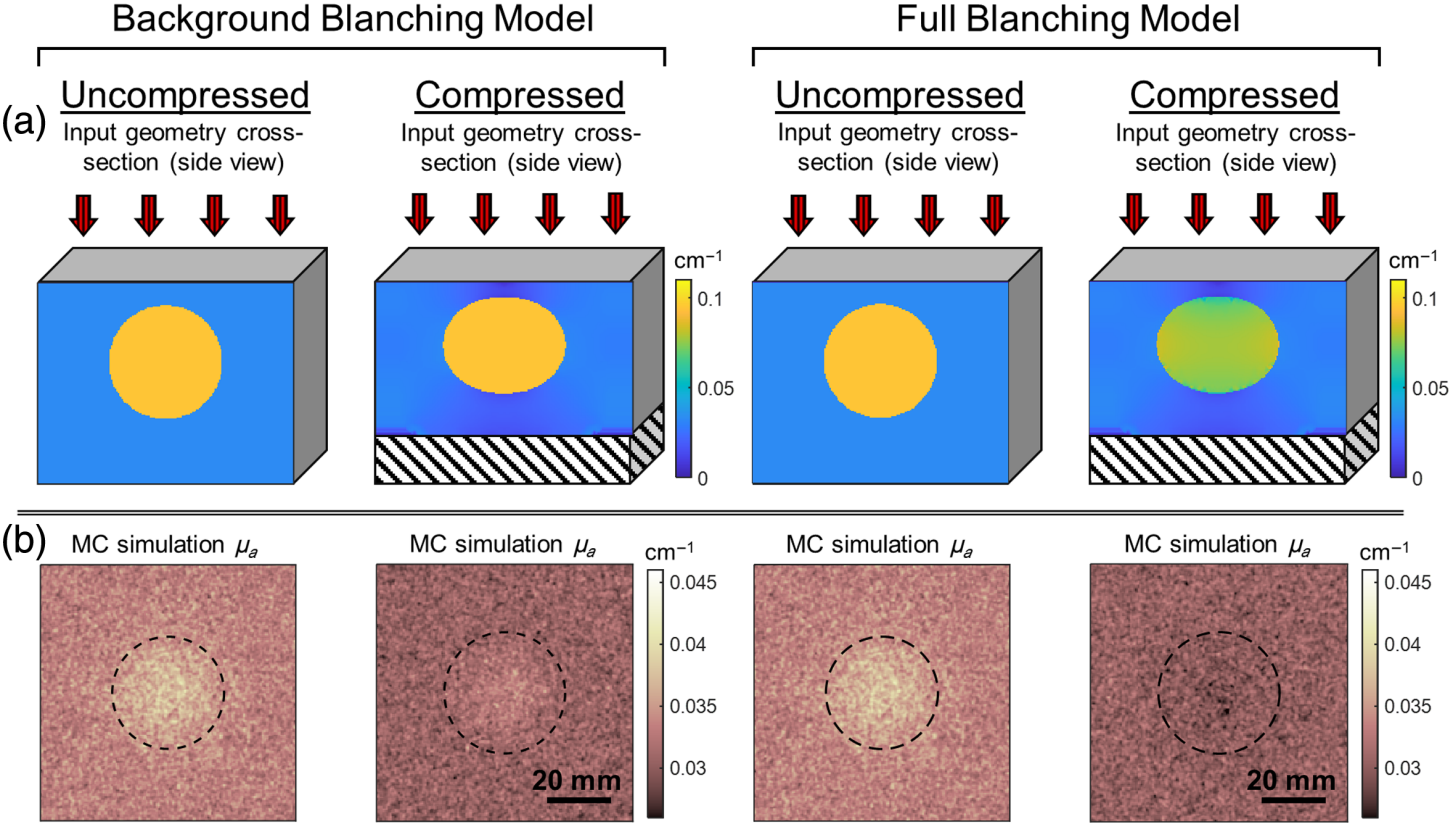
Summary of the two tissue blanching models, in which compression decreases $\mu_{a}$ of the inclusion, resulting in a reduction of optical contrast for some conditions. (a) Illustration of the effect of compression on the input model with simulated tissue blanching. Color represents simulation input $\mu_{a}$. The black and white striped area indicates the phantom height reduction from compression. (b) Results of MC simulations on UC and compressed conditions. Dotted lines mark the outline of the inclusion projected onto the phantom surface.

In simulating tissue blanching with compression, the same compressed geometry is used as in the “no blanching” condition, with final optical properties calculated from hydrostatic stress according to the relation described in “processing of FEA output” above. Again, all figures refer to the results using 50.6  μM tHb for tumor and 17.6  μM tHb for healthy breast. Representative examples of the effect of compression in the “background blanching” and “full blanching” condition and the resulting changes to the MC-derived $\mu_{a}$ contrast are shown in Fig. 6. Inclusion $r_{0}$, $d_{0}$, and SR are the same as in Fig. 4. Figure 6(a) shows the effect of compression on both the inclusion geometry cross-section and the optical property distribution of the inclusion and background.

In the solid phantom condition, optical contrast was found to be dependent on the final geometry alone. Although the SR in that condition influenced the shape change of the inclusion with compression (and thus influenced r and d), its value was not independently associated with final optical contrast. Conversely, in the “tissue blanching” condition, the SR strongly influenced optical contrast through its action on tHb reduction, and r and d alone described a comparatively small portion of the variance in contrast. As was done for the solid phantom condition, a surface of best fit of the form given in Eq. (3) was fit for the prediction of contrast from r and d. The equation of the best-fit surface was $z=(0.160-1.738r)\cdot e^{-0.1815d}-1.722$. This surface was a poor fit, failing to describe much of the variation in contrast, with $R^{2}=0.182$.

In the “background blanching” condition, compression has the effect not only to flatten the inclusion and decrease depth but also to reduce $\mu_{a}$ of the background, particularly the region of background directly above the inclusion. Although reduction in depth and flattening of the inclusion would act to increase optical contrast, the reduction in $\mu_{a}$ above the inclusion acts to decrease optical contrast. Figure 6(b) shows the combined influence of these factors on the simulation $\mu_{a}$ results, showing a decrease in optical contrast with compression. In the “full blanching” condition, the same reduction of $\mu_{a}$ above the inclusion occurs, and the inclusion itself also experiences a reduction in $\mu_{a}$. These factors result in a reduction of contrast with compression that is more pronounced than that of the “background blanching” condition.

### 3.3 Compression-Induced Contrast Change for all Conditions

In the “no blanching” condition, the vast majority of simulations experienced an increase in optical contrast from compression, across all sizes, depths, and SRs. In the two tissue blanching conditions, some simulations exhibited a decrease in contrast induced by compression, as demonstrated by the examples in Fig. 6. For simulations experiencing a compression-induced decrease in contrast, some maintained positive, though reduced, contrast values mid-compression, whereas others exhibited negative contrast values mid compression, where $\mu_{a}$ above the inclusion was lower than the background value. The percentage of simulations exhibiting contrast increase, contrast decrease that remained positive compression, and negative mid-compression contrast is reported in Table 3.

**Table 3:** Percentage of simulations falling into three categories based on the effect of compression on the contrast value.

SR	Contrast increased with compression (%)	Contrast reduced, remained positive (%)	Contrast became negative (%)
“No blanching” condition			
1	95	5	0
4	100	0	0
7	100	0	0
10	95	5	0
“Background blanching” model			
1	80	15	5
4	30	40	30
7	30	30	40
10	20	35	35
“Full blanching” model			
1	65	30	5
4	0	40	60
7	0	30	70
10	0	25	75

Results are separated into sub-tables for each of the three blanching models. For each table row, n=20 (five inclusion depths and four inclusion sizes).

**Fig. 7:**
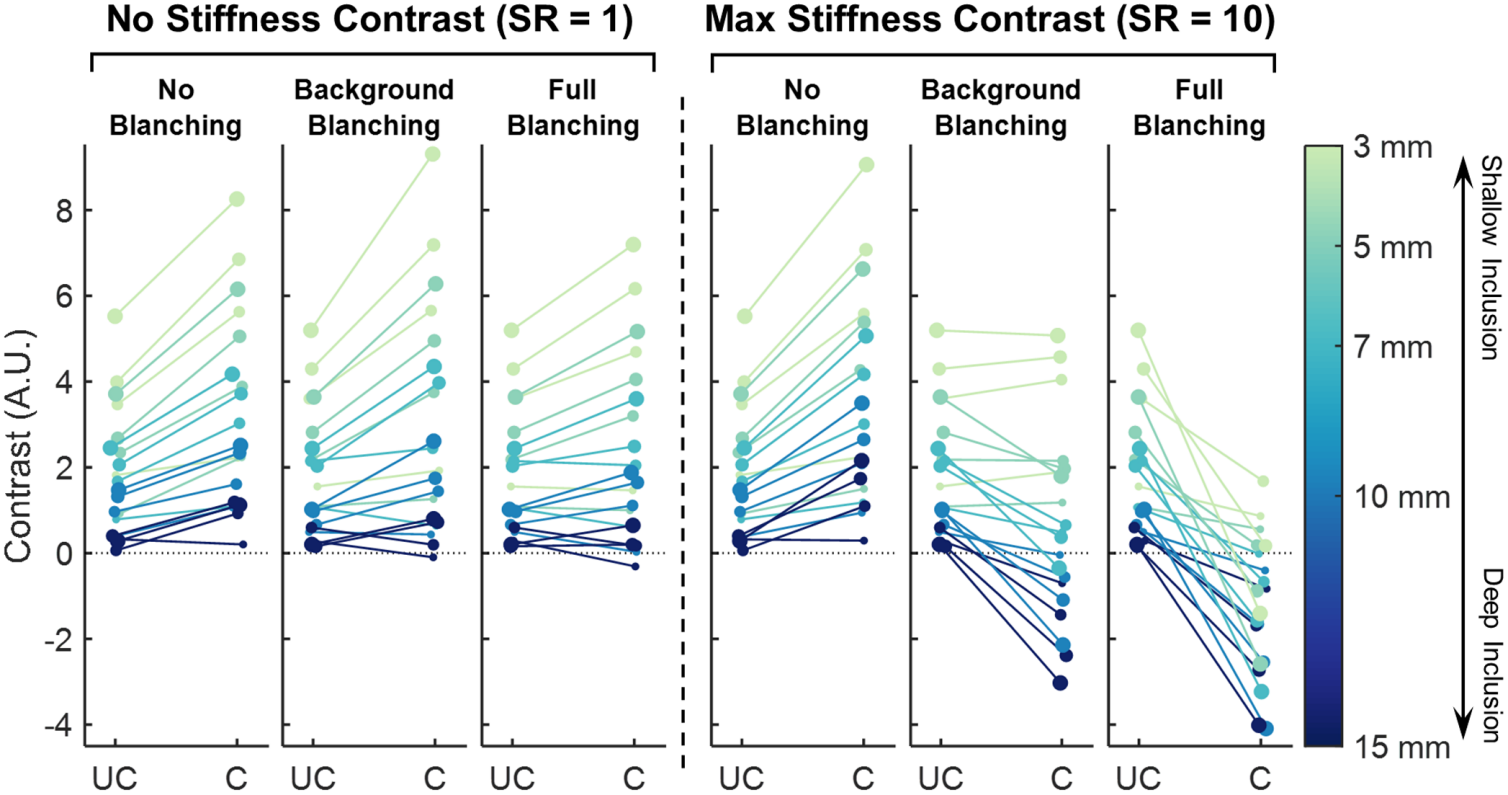
Measured contrast for uncompressed (UC) and compressed (C) cases, shown for the two extremes: SR=1 and SR=10. Initial inclusion depth is indicated by color, and inclusion radius is indicated by marker size. Plot colors adapted from Ref. 54.

Optical contrast before and during compression are illustrated for the two extremes of stiffness ratio, SR=1 and SR=10, in Fig. 7. For comparison with other work, these values of contrast have also been converted to the T/N ratio using the projection of the inclusion boundary onto the surface as the “tumor” ROI and image margins greater than 45 mm from inclusion epicenter as the “normal” ROI. For UC simulations, the T/N values range from ∼1 to 1.2 (0% to 20% increase in $\mu_{a}$) and after compression values range from ∼0.9 to 1.3 (10% decrease to 30% increase) depending on the blanching model used.

This reduction in contrast that occurs at high SRs is explained by the greatest hydrostatic stress (and thus greatest tHb reduction) occurring in the region directly above the inclusion, in which the soft background material is squeezed between the stiff inclusion and the compression plate. A greater reduction in tHb over the inclusion than in the surrounding areas serves to offset the contrast from the high tHb content of the inclusion itself. In the “full blanching” conditions, there can also be significant tHb reduction of the inclusion top surface as well, contributing to negative contrast in the compressed state.

To assess how the initial simulation parameters of SR, depth ($d_{0}$), and radius ($r_{0}$) affect the contrast change with compression, Pearson correlations were calculated for three variables and the difference between final and initial contrast for the three blanching models. These values are reported in Table 4. In the “no blanching” condition, the initial radius exerts the most effect on contrast change, with large inclusions gaining the most contrast from compression, followed by initial inclusion depth, with shallow inclusions gaining the most contrast from compression. In the “background blanching” model, SR and depth are negatively correlated with contrast change, that is, shallow inclusions with low stiffness are associated with a gain of contrast, whereas stiffer and deeper inclusions are associated with a reduction in contrast. In these cases, though inclusions are brought closer to the surface and blanching does not occur in the inclusion itself, the effect is outweighed by the blanching of the background material above the inclusion. Due to the high sensitivity of SFDI to superficial tissue, this blanching above the inclusion results in a decrease in contrast with compression for stiff and deep inclusions. When considering size in the “background blanching” condition, a large inclusion radius results in a wider range of outcomes in contrast change, whereas small radius inclusions experienced only small contrast changes, be they positive or negative. Finally, in the “full blanching” model, stiffness, as well as the inclusion radius, is again negatively correlated with change in contrast, with larger inclusions experiencing the most contrast reduction from compression. However, it was noted that, as for the “full blanching” condition, the correlation of radius with contrast change is reversed when only SR=1 is considered, that is, for SR=1, larger inclusions experienced a more positive change in contrast, whereas for all other SR, large inclusions experienced a more negative change in contrast. It can be said for both “background blanching” and “full blanching” models that a larger radius is associated with a higher magnitude of contrast change, the sign of which is determined by SR and, to a lesser extent, depth.

**Table 4:** Pearson correlation of initial simulation properties of SR, inclusion depth, and inclusion radius on contrast change from compression.

	“No blanching” condition		“Background blanching” model		“Full blanching” model	
	Correlation	p-value	Correlation	p-value	Correlation	p-value
SR	0.11	0.35	−**0.61**	**<0.001**	−**0.68**	**<0.001**
Depth ($d_{0}$)	−**0.53**	**<0.001**	−**0.50**	**<0.001**	−0.01	0.91
Radius ($r_{0}$)	**0.74**	**<0.001**	−0.12	0.30	−**0.42**	**<0.001**

Correlations with p-value<0.05 are indicated in bold.

When simulations are repeated with tHb concentration in the tumor voxels increased from 50.6 to 63.3  μM tHb for the “full blanching” condition, the trends in contrast change are found to be the same as those in Fig. 7, with SR exerting the greatest effect on contrast change. When the 63.3  μM simulations are compared directly with the 50.6  μM simulations, the increase in tumor tHb appears to have little effect on the trend in contrast change from compression in the “full blanching” model (not shown).

## 4 Discussion

In the “no blanching” condition, we examine the effect of inclusion size and depth on optical contrast. We confirm that, with optical properties of the inclusion and background held consistent, contrast is determined primarily on the final depth and shape of the inclusion top edge. This indicates that, for this measure of optical contrast, a compressed ellipsoidal inclusion is effectively indistinguishable from a spherical inclusion of radius equal to the ellipsoid top surface radius of curvature. In characterizing change in contrast based on initial parameters, the most gain in contrast was found for large inclusions and for initially shallow inclusions. The high contrast gain for large inclusions is explained by the idea that, for a given depth reduction, a larger mass of inclusion voxels is brought closer to the surface, resulting in a larger gain. Though deep inclusions experienced a greater absolute reduction in depth than initially shallow inclusions, these depth changes amounted to smaller contrast gains than shallow inclusions due to the exponential relationship between depth and contrast. When tHb content within the tumor is increased, the expected responses of greater baseline contrast and greater increase of contrast are observed. The relationship with stiffness is more complex, as stiff inclusions experienced a greater depth reduction than softer inclusions but had less tendency for the top surface to deform and become flatter. These two tendencies worked to counteract each other, and the SR exerted very little effect on contrast change.

Though these results represent a greatly simplified model, we may still consider these findings in terms of expected trends in a tumor during response to NAC, and we can predict that a tumor that becomes smaller would exhibit lower initial contrast and lower contrast gain from compression than prior to therapy, and a reduction in tumor tHb would exhibit the same effect. A reduction in tumor stiffness is not expected to have a large effect on initial contrast or contrast gain, except in cases in which the tumor is still quite large and quite deep, which might result in a smaller contrast gain. Thus, we can hypothesize that lower initial contrast and reduced contrast gain from compression could indicate a combination of tumor shrinkage and tHb reduction without being inconsistent with loss of tumor stiffness.

When a change in tHb in response to compression is modeled in the “background blanching” and “full blanching” conditions, the final geometry (depth and radius of curvature) is not sufficient for the predication of compressed contrast due to the strong effect of SR on ΔtHb. The relationship between initial inclusion properties and contrast change with compression is also more complex, with the SR exhibiting the greatest effect in both blanching models. The soft material superficial to stiff inclusions experienced significant hydrostatic stress, and thus a reduction of tHb, in this volume region. Due to the high sensitivity to superficial tissue of SFDI, this region of reduced tHb resulted in lower contrast between inclusion and background regions than seen before compression. Although this reduction in contrast from compression is a potential concern for clinical use of this imaging method, it is also possible that the information can be gleaned from this effect to its dependence on tumor size and stiffness.

Although SR=1 experiences a small positive contrast gain from compression, all other SRs experience a reduction in contrast, with a higher magnitude associated with higher stiffness. This reduction is also more pronounced in the “full blanching” condition as the surface of the inclusion also undergoes a reduction in tHb. After the SR, the initial inclusion depth shows the greatest correlation with contrast change in the “background blanching” model, with deep inclusions experiencing the most contrast reduction. In the “full blanching” condition, large inclusions experience more contrast reduction for SR values above one. More sophisticated mechanical modeling as well as *in vivo* imaging of breast tumors under compression using SFDI will be necessary to validate these trends. An increase in baseline tHb concentration within the tumor was not observed to have a significant effect on contrast gain in the “full blanching” condition.

Limitations of this study include the very low compression pressure used. The reaction force to the applied displacement was ∼0.5  N, compared with an applied force of ∼17  N by Sajjadi et al.^7^ That study involved whole-breast compression with a larger contact area than that used here, but any reasonable estimate of contact area yields a significantly higher pressure than that used here. Stronger compression likely would have induced a larger change in lesion depth, as well as different behavior in tissue blanching. For stiff inclusions (SR>1), the highest stresses were always experienced directly above the inclusion, and thus, the highest reduction in tHb happened in this location. The surrounding background material experienced lower stresses and thus a lower reduction in tHb. Negative contrast in many cases arose from the fact that blanching of the background surface occurred primarily above the inclusion with only small changes in tHb occurring further from the inclusion. This localized blanching of the surface served to offset the high tHb concentration of the inclusion, resulting in near-zero or negative final contrast. As we modeled tissue blanching to plateau at 20 mmHg, we would expect greater compression pressure to increase the area of the background that is experiencing the maximum tHb reduction. Higher pressure would result in maximal blanching of much of the background material surface, rather than only the region on top of the inclusion. With optical properties of the background surface closer to uniform in x and y, the high tHb content of the inclusion may still produce a positive contrast under these conditions.

At least one study reports a decrease in breast tissue $\mu_{s}^{\prime }$ resulting from compression,^55^ but the effect of tissue compression on scattering properties is generally not well characterized in the literature. This work assumed that $\mu_{s}^{\prime }$ remains constant during compression, and further research is needed to account for possible changes in scattering during compression.

Although this simplified model accounts for spatially varying ΔtHb induced by compression, the effect on time-varying hemodynamic changes is not covered by this work, nor are any changes in tissue oxygen saturation in response to compression. The effect of applied pressure on breast hemodynamics has been modeled in a simulation study by Nioka et al.,^56^ and further research in this area would be very useful. Utilizing diffuse optical tomography, differential changes in tissue oxygen saturation, as well as the time-course of changes in tHb, have been demonstrated between tumor and healthy breast in response to compression.^7^^,^^23^ A greater decrease of oxygen saturation was observed in the tumor, and as the pressure decreased due to viscoelastic relaxation (with displacement held constant), a persistent reduced tHb level was observed in the tumor in contrast to a gradual recovery of tHb in the normal tissue. This differential response was shown to normalize in patients responding to NAC.^7^

In addition, the viscoelastic character of breast tissue was not modeled. The tissue was modeled as a solid material with hydrostatic stress being used to determine tissue blanching; however, tissue consists of a solid porous structure containing interstitial fluid, and future work should directly consider the effect of applied pressure of interstitial fluid pressure.^57^

Compression-induced hemodynamics represent a complex interplay between blood inflow and outflow and local metabolic oxygen demand. Differential hemodynamic responses likely arise from mechanical differences;^5^^,^^41^^,^^58^ increased metabolic activity within the tumor;^4^^,^^59^ abnormalities in the vascular network, including disorganized and tortuous blood vessels;^60^^,^^61^ and increased interstitial fluid pressure within the tumor.^62^^,^^63^ Though not directly comparable with compression-induced changes, hemodynamic changes in response to breath-holding have also been shown to differ between tumor and healthy breast,^64^ and normalization of these differences can be used for NAC response prediction,^6^ further highlighting the value of tracking time-varying changes in hemoglobin concentrations for breast cancer therapy monitoring. A more complex dynamic model would be needed to predict how these hemodynamics will be evident in SFDI.

## 5 Conclusion

In this work, combined mechanical and optical simulations were used to show how attributes of a breast tumor-mimicking inclusion influenced measured contrast with SFDI and tissue compression. The two tissue blanching models used in this study predicted that compression of a stiff tumor would result in a reduction of contrast between tumor and normal tissue due to squeezing the healthy tissue superficial to the tumor, an effect of higher magnitude for large and very stiff tumors. By contrast, the “no blanching” model predicted that contrast will be gained from compression across all inclusion parameters, though this model is of limited applicability to *in vivo* imaging. Though no definitive conclusion may be reached at this time and much additional research is needed, this work represents a first step toward the potential to use SFDI with local tissue compression for tracking changes in breast tumors such as reduction in size and reduction in stiffness.

## Data Availability

Data and code used to generate results and figures are available on Code Ocean, https://doi.org/10.24433/CO.4469356.v1.
